# A Novel Predictor Compared to the Model for End-Stage Liver Disease (MELD) and Child-Turcotte-Pugh (CTP) Scores for Predicting 30-Day Mortality in Patients With Liver Cirrhosis

**DOI:** 10.7759/cureus.81446

**Published:** 2025-03-30

**Authors:** Ni Nyoman Gita Kharisma Dewi, I Ketut Mariadi, Ni Luh Putu Yunia Dewi, Kadek Mercu Narapati Pamungkas, Putu Itta Sandi Lesmana Dewi, Dwijo A Sindhughosa

**Affiliations:** 1 Division of Gastroenterology and Hepatology, Centre Research for Alimentary and Hepatobiliary System, Denpasar, IDN; 2 Division of Gastroenterology and Hepatology, Department of Internal Medicine, Udayana University, Denpasar, IDN; 3 Division of Gastroenterology and Hepatology, Department of Internal Medicine, Ngoerah Central General Hospital, Denpasar, IDN

**Keywords:** 30-day mortality, ctp and meld score, liver cirrhosis (lc), predictor of mortality, prognostic predictor

## Abstract

Background

Liver cirrhosis (LC) is characterized by the development of fibrosis and nodules within the liver, leading to progressive liver dysfunction. The mortality rate associated with LC has consistently remained high over the years. Prognostic tools such as the Child-Turcotte-Pugh (CTP) score and the Model for End-Stage Liver Disease (MELD) score are commonly used to predict mortality and assess the severity of LC. Both scoring systems rely on laboratory parameters, including serum albumin, total bilirubin, and international normalized ratio (INR) levels. However, the CTP score interpretation can be variable, and INR testing is not routinely performed in many clinical settings, which may limit its utility. This study aims to assess the MELD and CTP scores, along with a new predictive tool, in estimating 30-day mortality for patients with LC.

Methodology

This retrospective cohort study focuses on patients diagnosed with LC at Ngoerah Central General Hospital. Physical examination data and laboratory ratios, including neutrophil-to-lymphocyte ratio (NLR), aspartate transaminase (AST) to alanine transaminase (ALT) ratio (de Ritis), neutrophil-to-lymphocyte-to-albumin (NLA) ratio, albumin bilirubin index (ALBI), and blood urea nitrogen-to-albumin ratio (BAR), were collected from medical records. Optimal cutoff values were established using receiver operating characteristic (ROC) curves. Survival analysis was performed using the Kaplan-Meier method, and multivariate Cox regression was employed to determine the hazard ratio (HR) for each variable that was statistically significant as a predictor of 30-day mortality.

Results

In this study, a total of 140 samples were analyzed. Kaplan-Meier analysis revealed that hepatic encephalopathy (HE) met the criteria, while interaction analysis testing was required for other variables. Results from the multivariate Cox regression interaction model showed that ALBI-HE (HR = 1.743, 95% confidence interval [CI] 1.102-2.759, *P* = 0.018), BAR-HE (HR = 0.577, 95% CI 0.367-0.905, *P* = 0.017), and NLA-HE (HR = 0.332, 95% CI 0.195-0.563, *P* < 0.001) were significant independent predictors of 30-day mortality in LC. CTP, MELD, NLR, and de Ritis did not demonstrate statistical significance. ALBI-HE emerged as the strongest predictor based on its HR.

Conclusions

ALBI-HE, BAR-HE, and NLA-HE have emerged as novel predictors for assessing 30-day mortality in LC. ALBI-HE is the strongest predictor of 30-day mortality in LC.

## Introduction

Liver cirrhosis (LC) is a chronic liver disease with an estimated prevalence of 2.2 million cases in the United States and an average mortality rate of 21.9 per 100,000 individuals. Cirrhosis is characterized by the presence of fibrotic tissue, which results from various chronic liver conditions. The majority of cases (approximately 45%) are attributed to alcohol-related liver disease. The pathogenesis of cirrhosis involves chronic inflammation, leading to the replacement of healthy liver parenchyma with fibrotic tissue and regenerative nodules, ultimately causing complications such as portal hypertension [1-2]. 

LC can lead to complications such as hepatocellular carcinoma (HCC) and hepatic decompensation, characterized by the development of ascites, hepatic encephalopathy (HE), and variceal bleeding. These complications contribute to increased mortality, which was estimated at 2.4% globally in 2019. The total number of deaths from LC worldwide was approximately 1,472,000 in 2019, representing a 10% increase compared to 2010. The highest mortality rates were reported in Southeast Asia, accounting for 443,000 deaths [3].

Currently, the tools used to estimate the prognosis of patients with LC include the Child-Turcotte-Pugh (CTP) score and the Model for End-Stage Liver Disease (MELD) score. While the CTP score is user-friendly, it relies on subjective assessments of ascites and encephalopathy severity, leading to considerable variability in interpretation. Both scoring systems also depend on laboratory parameters such as albumin, total bilirubin, and international normalized ratio (INR) levels. However, INR testing is not routinely performed in many clinical settings, which may limit the widespread applicability of these tools.

Recent studies have identified additional markers, such as the neutrophil-to-lymphocyte ratio (NLR), which are associated with mortality in LC and may serve as prognostic indicators for survival. Building on this evidence, this study aims to identify laboratory and physical examination markers that can predict the one-month survival rate of patients with LC. The goal is to assess whether these markers offer predictive value comparable to or exceeding that of the established CTP and MELD scores.

This article was previously presented as a meeting abstract at the Asian Pacific Digestive Week (APDW) 2024 on November 24, 2024.

## Materials and methods

Population and study design

This retrospective cohort study included 140 patients treated at Ngoerah General Hospital between July 2020 and January 2024. Eligible participants were adults aged 18 years or older, of any gender, diagnosed with LC, and had provided informed consent. Patients with malignancies, including primary liver cancer, were excluded from the study. All participants underwent a complete blood count and detailed liver function tests, including liver enzymes, serum albumin, total bilirubin, direct and indirect bilirubin, and coagulation factors.

Demographic, clinical, and laboratory values in this study

The study included demographic data such as age and gender, along with the etiology of LC, clinical manifestations, and complete blood count results, all obtained from medical records. Laboratory results were deemed normal if they fell within the following ranges: white blood cell (WBC; 4.1-11.0 x 10³/μL), platelets (140-440 x 10³/μL), hemoglobin (12-16 g/dL), albumin (3.40-4.80 g/dL), blood urea nitrogen (BUN; 8.00-23.00 mg/dL), and sodium (136-145 mmol/L).

Indicators related to LC and laboratory ratios

We identified five laboratory test ratios associated with the survival rate of LC and compared them with the MELD and CTP scores, which are commonly used to predict the prognosis of patients with LC. CTP and MELD scores were calculated using physical examination findings and laboratory results, such as creatinine, bilirubin, and INR. The five laboratory ratios included the neutrophil-basophil count, albumin-bilirubin index (ALBI), NLR, neutrophil-to-lymphocyte-to-albumin ratio (NLA), the serum glutamate oxaloacetate aminotransferase (SGOT) to serum glutamate pyruvate aminotransferase (SGPT) ratio (de Ritis), and the blood urea nitrogen-to-albumin ratio (BAR). A receiver operating characteristic (ROC) analysis was conducted to determine the cutoff values for all parameters: CTP score ≥ 9.5, MELD score ≥ 14.5, de Ritis ≥ 1.685, ALBI ≥ -1.22, BAR ≥ 9.11, NLR ≥ 5.5, and NLA ≥ 2.16.

Statistical data analysis 

Data were collected and analyzed using IBM SPSS software version 29.0 (IBM Corp., Armonk, NY). Descriptive analysis was performed for all variables. Numerical variables that were normally distributed were expressed as mean ± standard deviation, while non-normally distributed data were presented as median (minimum-maximum). Nominal variables were presented as counts and percentages. Bivariate analysis of categorical data was conducted using the Chi-square test, with statistical significance defined as a *P*-value < 0.05. Dependent variables with a *P*-value < 0.25 were further analyzed using multivariate binary logistic regression, with significance set at *P* < 0.05.

For all dependent variables deemed significant, proportional hazard (PH) assumptions were checked. PH assumptions were evaluated using Kaplan-Meier curves. Variables that met the PH assumption were further analyzed using multivariate Cox regression to determine the hazard ratio (HR). However, variables that did not meet the PH assumption could still be included in the multivariate Cox regression if they were considered clinically relevant, using interaction or stratification models. The results of the multivariate Cox regression were considered significant if the *P*-value was <0.05, and the strength of the association was assessed based on the HR. 

Ethical clearance

This research is part of an approved investigation by the Research Committee of the Faculty of Medicine at Udayana University/Ngoerah General Hospital, with ethical clearance number 92/UN14.2.2.VIII.17/PD/2024.

## Results

A total of 140 patients were included in this retrospective cohort observational study. The majority were male (98, 70%), with a mean age of 53.46 ± 10.64 years. The most common comorbidities were renal disease (40, 28.6%) and diabetes mellitus (34, 24.3%). Hepatitis B virus (HBV) was the primary cause of LC, accounting for 66 (47.1%) cases. Poor prognosis, indicated by MELD scores ≥14.5 and CTP scores ≥9.5, was observed in 67 (47.9%) patients. Furthermore, 58 (41.4%) patients died within 30 days. Detailed data on the sample characteristics are presented in Table 1.

**Table 1:** Characteristics of the study cohort. HBV, hepatitis B virus; HCV, hepatitis C virus; MELD, model for end-stage liver disease; CTP, Child-Turcotte-Pugh; AST, aspartate aminotransferase; ALT, alanine aminotransferase; INR, international normalized ratio; APTT, activated partial thromboplastin time; de Ritis, AST/ALT ratio; NLR, neutrophil-to-lymphocyte ratio; NLA, neutrophil-lymphocyte-albumin ratio; ALBI, albumin-bilirubin index; BAR, blood urea nitrogen-to-albumin ratio

Parameters	*n* = 140
Age (mean ± SD)	53.46 ± 10.64
Gender, *n* (%)	
Man	98 (70)
Woman	42 (30)
Etiology, *n* (%)	
HBV	66 (47.1)
HCV	40 (28.6)
Alcohol	1 (0.7)
Unknown	33 (23.6)
MELD, *n* (%)	
≥14.5	67 (47.9)
<14.5	73 (52.1)
CTP, *n* (%)	
≥9.5	67 (47.9)
<9.5	73 (52.1)
Mortality in 30 days, *n* (%)	58 (41.4)
Past medical history, *n* (%)	
Diabetes mellitus	34 (24.3)
Hypertension	11 (7.9)
Renal disease	40 (28.6)
Stroke	1 (0.7)
Spontaneous bacterial peritonitis	13 (9.3)
Physical examination, *n* (%)	
Ascites	93 (66.4)
Hepatic encephalopathy	58 (41.4)
Laboratory result, median (range)	
AST	51 (12.1-4780)
ALT	29.9 (5-1329)
Bilirubin total	2.4 (0.4-146)
INR	1.4 (0.88-239)
APTT	33.2 (10.6-664)
Albumin	2.7 (0.3-36)
Laboratory ratio, *n* (%)	
de Ritis	
≥1.685	69 (49.3)
<1.685	70 (50)
ALBI	
≥-1.22	65 (46.4)
	75 (53.6)
BAR	
≥9.11	65 (46.4)
<9.11	75 (53.6)
NLR	
≥5.5	61 (43.6)
<5.5	79 (56.4)
NLA	
≥2.16	64 (45.7)
<2.16	76 (54.3)

Bivariate analysis was performed to identify variables associated with 30-day mortality in patients with LC. Variables with a *P*-value < 0.25 were included in the subsequent multivariate analysis. Of the 16 variables tested, 12 met the inclusion criteria for multivariate analysis. The results revealed that six variables were statistically significant predictors of 30-day mortality in patients with LC: HE (*P* < 0.001), renal disease (*P* = 0.003), ascites (*P* = 0.010), ALBI score (*P* = 0.047), hypertension (*P* = 0.005), and NLR (*P* < 0.001). Comprehensive data on the bivariate and multivariate analyses are detailed in Tables 2-3.

**Table 2:** Results of bivariate analysis. Analyzed by chi-square test. SBP, spontaneous bacterial peritonitis; MELD, Model for End-Stage Liver Disease; CTP, Child-Turcotte-Pugh; de Ritis, AST/ALT ratio; NLR, neutrophil-to-lymphocyte ratio; NLA, neutrophil-lymphocyte-albumin ratio; ALBI, albumin-bilirubin index; BAR, blood urea nitrogen-to-albumin ratio; CI, confidence interval

Parameter	*P*-value	95% CI
Gender	0.002	1.59-8.46
Age	0.63	0.60-2.32
Hypertension	0.103	0.6-1.39
Diabetes mellitus	0.67	0.38-1.85
Renal disease	<0.001	2.45-12.12
Stroke	0.40	1.49-1.97
SBP	0.006	1.44-20.93
Hepatic encephalopathy	<0.001	5.69-28.83
Ascites	<0.001	1.76-8.86
ALBI	<0.001	2.15-9.07
BAR	<0.001	2.78-12.20
NLR	<0.001	2.36-10.06
de Ritis	0.35	0.70-2.72
NLA	<0.001	5.99-31.03
CTP	<0.001	3.25-14.76
MELD	<0.001	2.20-9.32

**Table 3:** Results of multivariate analysis. Analyzed by multivariate binary logistic regression. NLR, neutrophil-to-lymphocyte ratio; NLA, neutrophil-lymphocyte-albumin ratio; ALBI, albumin-bilirubin index; BAR, blood urea nitrogen-to-albumin ratio; CI, confidence interval

Parameter	*P*-value	95% CI
Step 4		
Gender	0.09	0.82-13.28
Hepatic encephalopathy	<0.001	5.92-95.08
Renal disease	0.012	1.46-21.09
Ascites	0.01	1.62-33.71
ALBI	0.06	0.07-1.08
BAR	0.23	0.61-7.68
NLR	0.14	0.02-1.73
Hypertension	0.01	0.004-0.50
NLA	0.001	4.99-877.22
Step 5		
Gender	0.083	0.85-13.74
Hepatic encephalopathy	<0.001	6.09-94.33
Renal disease	0.003	1.90-25.32
Ascites	0.010	1.61-32.20
ALBI	0.047	0.07-0.98
NLR	0.055	0.015-1.047
Hypertension	0.005	0.003-0.367
NLA	<0.001	10.99-1426.79

Survival analysis 

The survival analysis began with an assessment of the PH assumption, evaluated using Kaplan-Meier curves. Among the variables identified as significant in the multivariate binary logistic regression, only HE met the PH assumption. Variables that did not meet the PH assumption were included in the multivariate Cox regression analysis if they were considered to influence mortality in patients with LC.

Previous studies have shown that laboratory ratios, including the de Ritis ratio, ALBI, BAR, NLR, and NLA, are significantly associated with mortality in LC [4-6]. Glisic et al. demonstrated that the ALBI score, NLR, and NLA possess prognostic value in predicting 30-day mortality in patients with LC (*P* < 0.05) [4]. Similarly, Zhang et al. identified the BAR as a potential prognostic tool for predicting mortality in patients with HBV-related decompensated cirrhosis, as evidenced by an area under the curve (AUC) of 0.752 (specificity, 77.3%; sensitivity, 74.2%; *P* < 0.001) [6]. Additionally, MELD and CTP scores, which are commonly used as prognostic markers, were included in the multivariate Cox regression analysis. These variables, identified in prior studies as influencing mortality in patients with LC, were incorporated into the Cox regression using interaction models.

**Figure 1:**
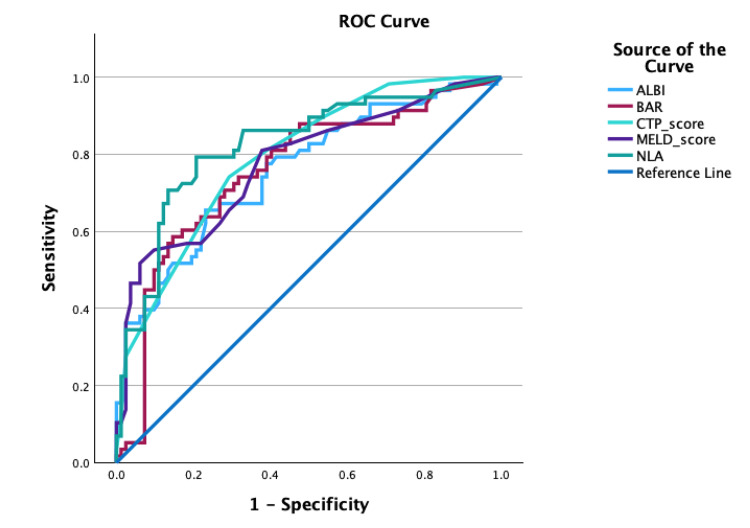
ROC analysis of predictors for 30-day mortality in patients with liver cirrhosis. ALBI, albumin-bilirubin index; BAR, blood urea nitrogen-to-albumin ratio; NLA, neutrophil-to-lymphocyte-to-albumin ratio; MELD, Model for End-Stage Liver Disease; CTP, Child-Turcotte-Pugh; ROC, receiver operating characteristic

**Figure 2:**
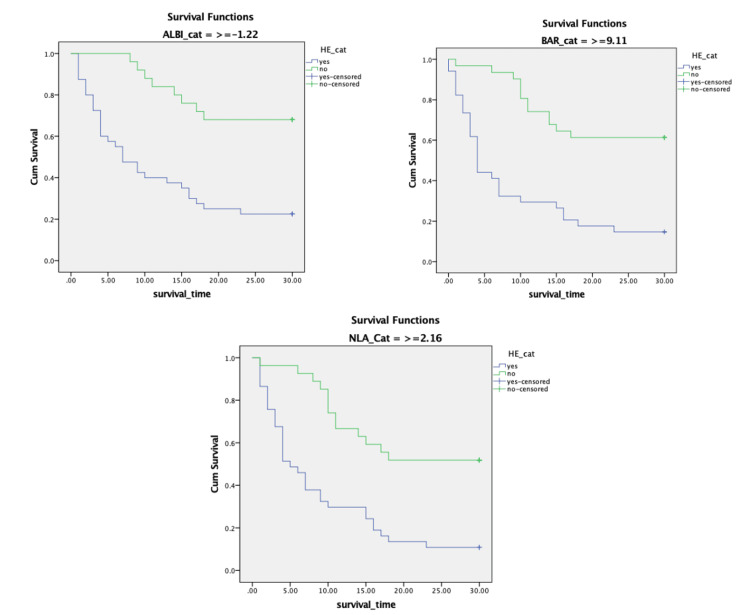
Kaplan-Meier survival curve of novel predictors for 30-day mortality in patients with liver cirrhosis. Kaplan-Meier survival curves predicting 30-day mortality in patients with liver cirrhosis based on ALBI levels (cutoff ≥ -1.22) and the presence of hepatic encephalopathy (log-rank test, 36.594; Df = 1; *P* < 0.001); BAR levels (cutoff ≥ 9.11) and the presence of hepatic encephalopathy (log-rank test, 45.162; Df = 1; *P* < 0.001); and NLA levels (cutoff ≥ 2.16) and the presence of hepatic encephalopathy (log-rank test, 33.971; Df = 1; *P* < 0.001). Df, degrees of freedom; BAR, blood urea nitrogen-to-albumin ratio; NLA, neutrophil-to-lymphocyte-to-albumin ratio; ALBI, albumin-bilirubin index

The results from the multivariate Cox regression interaction model revealed that ALBI-HE (HR = 1.743, 95% CI 1.102-2.759; *P* = 0.018), BAR-HE (HR = 0.577, 95% CI 0.367-0.905; *P* = 0.017), and NLA-HE (HR = 0.332, 95% CI 0.195-0.563; *P* < 0.001) were significant independent predictors of 30-day mortality in LC. Detailed data on the results of the multivariate Cox regression analysis are presented in Table 4. This study presents the AUC values for each predictive variable significantly associated with 30-day mortality in patients with LC, as illustrated in Figure 1. The ROC curve analysis demonstrates that NLA (AUC = 0.825, *P* < 0.001) exhibits the highest prognostic value compared to CTP (AUC = 0.793, *P* < 0.001), MELD (AUC = 0.777, *P* < 0.001), ALBI (AUC = 0.758, *P* < 0.001), and BAR (AUC = 0.757, *P* < 0.001). A more detailed interpretation of the ROC analysis is provided in Table 5. This study also presents the Kaplan-Meier curve for these predictors in Figure 2, demonstrating that all of these predictors meet the PH assumption.

**Table 4:** The result of the multivariate Cox regression interaction model. ALBI, albumin-bilirubin index; BAR, blood urea nitrogen-to-albumin ratio; NLA, neutrophil-to-lymphocyte-to-albumin ratio; HE, hepatic encephalopathy; CI, confidence interval; HR, hazard ratio

No.	Variable	HR	95% CI		*P*-value
			Lower	Upper	
Step 4					
1	ALBI-HE	1.822	1.143	2.905	0.012
2	BAR-HE	0.592	0.374	0.935	0.025
3	NLR-HE	1.292	0.769	2.170	0.333
4	NLA-HE	0.255	0.119	0.549	<0.001
5	MELD-HE	0.626	0.384	1.020	0.06
Step 5					
1	ALBI-HE	1.743	1.102	2.759	0.018
2	BAR-HE	0.577	0.367	0.905	0.017
3	NLA-HE	0.332	0.195	0.563	<0.001
4	MELD-HE	0.665	0.415	1.065	0.09

**Table 5:** Prognostic accuracies of predictors for 30-day mortality in patients with liver cirrhosis. AUC, area under the curve; ALBI, albumin-bilirubin index; BAR, blood urea nitrogen-to-albumin ratio; NLA, neutrophil-to-lymphocyte-to-albumin ratio; MELD, Model for End-Stage Liver Disease; CTP, Child-Turcotte-Pugh

	AUC	*P*-value	Cutoff value	Sensitivity	Specificity
NLA	0.825	<0.001	2.16	79.3	78
ALBI	0.758	<0.001	-1.22	67.2	68.3
BAR	0.757	<0.001	9.11	70.7	70.7
MELD	0.777	<0.001	14.5	69	67.1
CTP	0.793	<0.001	9.5	74.1	70.7

## Discussion

In this retrospective cohort study involving 140 patients with LC, the primary causes of cirrhosis were identified as HBV infection, followed by HCV infection. The average age of the study participants was 53.46 ± 10.64 years, with males predominating at 98 (70%). The severity of LC was assessed using the CTP and MELD scores. These findings align with epidemiological studies on LC. 

The characteristics of the study samples are consistent with previous epidemiological research on LC. For instance, Huang et al. reported that the most common causes of LC include HBV and HCV infections, alcohol-associated liver disease, and non-alcoholic fatty liver disease (NAFLD). Globally, HBV infection accounts for 42% of cases with LC, while HCV infection accounts for 21%. According to World Health Organization (WHO) data, HBV-related LC is most prevalent in the Western Pacific region (59%) and least prevalent in the Americas (5%) [3]. Furthermore, Liu and Chen highlighted that in 2017, the prevalence of compensated cirrhosis was 58.8%, while decompensated cirrhosis reached 60.3% in male patients, indirectly indicating a higher prevalence of LC among men [7].

The survival analysis began with an assessment of the PH assumption for six variables identified as significant in the multivariate analysis. Only HE met the PH assumption, while the other variables did not. However, laboratory ratios, such as the de Ritis ratio, NLR, ALBI, NLA, and BAR, were included in the multivariate Cox regression analysis along with HE using interaction tests, as these variables were clinically relevant based on findings from previous studies.

The results from the multivariate Cox regression interaction model revealed that ALBI-HE (HR = 1.743, 95% CI 1.102-2.759, *P* = 0.018), BAR-HE (HR = 0.577, 95% CI 0.367-0.905, *P* = 0.017), and NLA-HE (HR = 0.332, 95% CI 0.195-0.563, *P* < 0.001) were significant independent predictors of 30-day mortality in LC. This study successfully presents the ROC analysis of the three independent predictors of 30-day mortality in patients with LC, compared with the CTP and MELD scores. Based on the ROC analysis, NLA (AUC = 0.825, *P* < 0.001) demonstrates the highest prognostic value compared to CTP (AUC = 0.793, *P* < 0.001), MELD (AUC = 0.777, *P* < 0.001), ALBI (AUC = 0.758, *P* < 0.001), and BAR (AUC = 0.757, *P* < 0.001). However, according to the multivariate Cox regression interaction model, ALBI-HE emerged as the strongest predictor based on its HR. These three variables are valid predictors of 30-day mortality in LC when considered in the context of the pathogenesis of the disease. LC is associated with immune dysfunction, characterized by a combination of systemic inflammation and immune deficiency. This inflammatory state is driven by the release of pro-inflammatory cytokines, which enhance neutrophil production while suppressing lymphocyte activity, leading to an elevated NLR. Cirrhosis is characterized by moderate to severe systemic inflammation, evidenced by elevated levels of inflammatory cytokines, oxidative stress, and markers of activated neutrophils and macrophages in the blood and liver [8-12]. Systemic inflammation is further exacerbated by the translocation of bacterial products from the gut into the bloodstream, a process driven by intestinal dysbiosis, increased intestinal permeability, and impaired gut immunity [10,12-16]. Inflammatory markers have significant predictive value in this context, for example, elevated NLR serves as a predictor of short-term mortality [12,17,18].

In LC, the liver's ability to conjugate and excrete bilirubin into bile is impaired, leading to the accumulation of unconjugated or conjugated bilirubin in the bloodstream, ultimately resulting in hyperbilirubinemia. Additionally, liver dysfunction also impairs albumin synthesis, contributing to hypoalbuminemia. These physiological disruptions underpin the relevance of the identified predictors in assessing mortality risk in patients with LC [18].

The major causes of mortality in patients with LC are complications arising from the progression of the disease. Chronic liver injury, caused by factors such as alcohol use, hepatitis, or fatty liver disease, leads to liver scarring (fibrosis), which obstructs normal blood flow through the liver. This process is often exacerbated by an overactive or exaggerated immune response, as seen in conditions like non-alcoholic steatohepatitis (NASH) or chronic hepatitis C (CHC), where the replacement of hepatic parenchyma with scar tissue and vascular architectural distortion results in organ dysfunction [19]. Consequently, the obstruction of blood flow increases pressure in the portal vein, the primary vein supplying blood, resulting in portal hypertension. The elevated pressure forces blood to bypass the liver through collateral veins, leading to complications such as varices, ascites, and splenomegaly [18].

Portal hypertension also impairs effective blood flow to the kidneys, causing retention of water and sodium, which results in altered renal function, typically marked by an elevated BUN level [18]. Additionally, LC promotes the overgrowth of harmful bacteria in the gut, leading to gut inflammation, damage to the intestinal barrier, and increased ammonia production. Pathogenic bacteria and pathogen-associated molecular patterns (PAMPs) enter the bloodstream, activating the immune system and triggering brain inflammation, which contributes to the development of HE [20].

The findings of this study are consistent with previous research. The study by Riggio et al. demonstrated that the six- and 12-month probabilities of death were significantly higher in the HE group, at 34.3% (95% CI, 30.4%-37.5%) and 40.8% (95% CI, 38.4%-43.2%), respectively, compared to the no HE group, which had rates of 18.7% (95% CI, 14.8%-21.3%) and 26.3% (95% CI, 22.4%-28.5%) [21]. Similarly, the study by Glisic et al. found that MELD (*P* < 0.001) and NLA (*P* = 0.014) were reliable predictors of HE. Additionally, MELD (*P* = 0.001), NLR (*P* < 0.001), PLR (*P* = 0.014), ALBI (*P* = 0.003), and NLA (*P* < 0.001) were identified as valuable prognostic markers for predicting 30-day mortality in patients with cirrhosis [4].

The research by Zhang et al. also highlighted the significance of NLA as a novel biomarker for predicting prognosis in patients with alcoholic cirrhosis. They identified a cutoff value of 19.6 for NLA, which provided sensitivity and specificity values of 70.0% and 72.5%, respectively. From the ROC curve analysis, NLA was found to be a better predictor of 30-day mortality compared to the MELD, Maddrey’s discriminant function (MDF), and integrated MELD (i-MELD) scores in patients with alcoholic cirrhosis [22].

The study by Naqvi et al. assessed the ability of the ALBI score as a predictor of in-hospital mortality in patients with LC, particularly in the context of viral infections. The findings were consistent with the results of this study. ROC analysis revealed that the ALBI score had the largest AUC compared to the CTP and MELD scores, with an AUC of 0.852 (95% CI, 0.826-0.879; *P* < 0.001). The optimal cutoff value for ALBI was -0.832, with both sensitivity and specificity at 78.1%. In cirrhosis caused by non-viral infections, the AUC of ALBI was significantly larger than that of MELD (*P* = 0.013), while it was comparable to the CTP score (*P* = 0.067). Furthermore, there was no significant difference between the AUCs of CTP and MELD (*P* = 0.092) [23].

The study by Zhang et al. highlighted that multivariate analysis identified the BAR and MELD scores as independent predictors of 30-day mortality in patients with HBV-associated decompensated cirrhosis. Among the various prognostic scores assessed, BAR demonstrated the highest sensitivity (72.4%) compared to MELD, C-reactive protein-to-albumin ratio (CAR), D-dimer-to-albumin ratio (DAR), prothrombin time-International Normalized Ratio-to-albumin ratio (PTAR), and neutrophil count-to-albumin ratio (NAR), which had sensitivity values of 65.5%, 55.1%, 24.1%, 65.5%, and 55.2%, respectively. The MELD score showed the highest positive predictive value (PPV) at 54.3%, while both MELD and BAR exhibited the best negative predictive values (NPV) at 92.1% and 92.7%, respectively. When combined, BAR and MELD scores enhanced the sensitivity to 75.8%, specificity to 84.1%, and the AUC to 0.815 [6].

The limitation of this study is that it used a small sample size and data from only one center. The results obtained are still not representative of the broader population. To validate the findings, it is expected that future studies will use a larger sample size.

## Conclusions

The ALBI-HE, BAR-HE, and NLA-HE demonstrated significant predictive value. These findings suggest their potential as novel predictors for assessing 30-day mortality in patients with LC in this cohort study. However, further validation through multi-center studies is necessary to confirm their clinical applicability. 

The ALBI-HE provides a simple and practical approach, reducing variability in interpretation by categorizing hepatic encephalopathy as either absent or present, without using the graded classification of the CTP score. Additionally, ALBI-HE does not require INR measurements, unlike the CTP and MELD scores, making it a more accessible and user-friendly prognostic marker in clinical practice, particularly in regions where INR testing is not routinely performed.
